# Apple Pomace Consumption Favorably Alters Hepatic Lipid Metabolism in Young Female Sprague-Dawley Rats Fed a Western Diet

**DOI:** 10.3390/nu10121882

**Published:** 2018-12-03

**Authors:** Roy Chris Skinner, Derek C. Warren, Soofia N. Lateef, Vagner A. Benedito, Janet C. Tou

**Affiliations:** 1 Division of Animal and Nutritional Sciences, West Virginia University, Morgantown, WV 26506, USA; rcskinner@mix.wvu.edu (R.C.S.); dwarren2@mix.wvu.edu (D.C.W.); 2 Department of Chemical Engineering, West Virginia University, Morgantown, WV 26506, USA; snlateef@mix.wvu.edu; 3 Division of Plant and Soil Sciences, West Virginia University, Morgantown, WV 26506, USA; vagner.benedito@mail.wvu.edu

**Keywords:** apple pomace, NAFLD, Western diet, DGAT2, bile acids, food waste, sustainability

## Abstract

Apple pomace, which is a waste byproduct of processing, is rich in several nutrients, particularly dietary fiber, indicating potential benefits for diseases that are attributed to poor diets, such as non-alcoholic fatty liver disease (NAFLD). NAFLD affects over 25% of United States population and is increasing in children. Increasing fruit consumption can influence NAFLD. The study objective was to replace calories in standard or Western diets with apple pomace to determine the effects on genes regulating hepatic lipid metabolism and on risk of NAFLD. Female Sprague-Dawley rats were randomly assigned (*n* = 8 rats/group) to isocaloric diets of AIN-93G and AIN-93G/10% *w*/*w* apple pomace (AIN/AP) or isocaloric diets of Western (45% fat, 33% sucrose) and Western/10% *w*/*w* apple pomace (Western/AP) diets for eight weeks. There were no significant effects on hepatic lipid metabolism in rats fed AIN/AP. Western/AP diet containing fiber-rich apple pomace attenuated fat vacuole infiltration, elevated monounsaturated fatty acid content, and triglyceride storage in the liver due to higher circulating bile and upregulated hepatic DGAT2 gene expression induced by feeding a Western diet. The study results showed the replacement of calories in Western diet with apple pomace attenuated NAFLD risk. Therefore, apple pomace has the potential to be developed into a sustainable functional food for human consumption.

## 1. Introduction

One-third of apples harvested in the United States (USA) are processed into apple products [1]. Apple pomace is a byproduct of apple processing that includes: skin, stem, seeds, core, and calyx [2]. Apple pomace presents an environmental and public health issue due to its rapid spoilage and fermentation. Moreover, apple pomace disposal is expensive with annual costs being estimated at $10 million in the USA [3,4]. Yet, apple pomace is a rich source of various nutrients (e.g., phytochemicals, vitamins, dietary minerals), but it is particularly high in non-digestible carbohydrates and dietary fibers, indicating potential benefits for reducing metabolic dysfunction, such as non-alcoholic fatty liver disease (NAFLD) [5].

NAFLD is characterized by dysregulated lipid metabolism and liver steatosis. It is the most prevalent liver disease worldwide with reports of over 25% of the population having NAFLD [6]. Global prevalence in children is estimated to be between 7.6–34.2% [7]. Further, liver steatosis has also been diagnosed in non-obese patients [8]. Diets that are high in fat and sucrose, which characterize Western diets, have been shown to induce NAFLD [9,10]. High carbohydrate consumption has been linked to NAFLD progression by upregulating the expression of key gene transcription factors that are involved in hepatic de novo lipogenesis (DNL), such as sterol regulatory element-binding protein-1c (SREBP-1c) and carbohydrate response element binding protein (ChREBP) [11]. SREBP-1c and ChREBP stimulate fatty acid synthase (FAS) to catalyze the synthesis of saturated fatty acids (SFAs). In turn, SFAs can be desaturated to monounsaturated fatty acids (MUFAs) by stearoyl-CoA desaturase-1 (SCD-1). Promoting fatty acid esterification by diacylglycerol *O*-acyl transferase-2 (DGAT2) stimulates hepatic triglyceride synthesis that can contribute to hepatic steatosis and production of triglyceride-rich very low density lipoproteins (VLDLs), which characterizes NAFLD [12]. 

Currently, there are no approved drugs for the treatment of NAFLD; therefore, management of NAFLD relies on proper diet and lifestyle changes [13]. Despite a paucity of studies, apple pomace has been shown to reduce metabolic risk factors, such as hyperglycemia and dyslipidemia, providing rationale for apple pomace consumption to reduce NAFLD [14,15]. However, a potential concern is apple pomace’s fructose content. Excessive fructose consumption has been shown to promote NAFLD development and progression [16]. Previously, we reported growing female Sprague-Dawley rats consuming different fructose containing drinks for eight weeks promoted NAFLD, but had no significant effect on body weight when compared to the water control [17]. Therefore, the objectives of this study were to determine the effects of caloric substitution with apple pomace in a normal (standard diet) or Western diet on expression of genes regulating hepatic lipid metabolism and NAFLD risk in a rat model. Our study followed the suggested dietary advice of replacing calories in the diet with healthier food choices instead of dietary supplementation with a purified isolated nutrient [18]. Study results showed that apple pomace had no detrimental effects on hepatic lipid metabolism and liver health in rats consuming normal diets and attenuate features in the NAFLD spectrum of upregulated gene expression of triglyceride synthesis as well as liver steatosis induced in rats consuming a Western diet. Investigating whether apple pomace, a byproduct generated from apple processing, can be re-purposed as a functional food for human consumption has the potential to improve public health and food sustainability by providing an economical solution for reducing environmental pollution and costly waste disposal.

## 2. Materials and Methods

### 2.1. Animals and Diets

Weanling (age 22–29 days) female Sprague-Dawley rats (*n* = 32) were purchased from Harlan-Teklad (Indianapolis, IN, USA). Female rats were selected on the basis of their greater susceptibility to hepatic effects with increased carbohydrate consumption [19]. All animal procedures were approved by the Animal Care and Use Committee at West Virginia University and were conducted in accordance with the guidelines of the National Research Council for the Care of Laboratory Animals [20]. Rats were individually housed with cages kept in a room at constant temperature of 21 ± 2 °C with a 12 h light/dark cycle throughout the study.

Following seven-days acclimation, rats were randomly assigned (*n* = 8 rats/group) to four dietary groups consisting of: (1) AIN-93G, a standard purified rodent diet (AIN), (2) AIN-93G with 10% weight (g/kg) substituted with apple pomace (AIN/AP), (3) Western diet (45% fat, 33% sucrose by kcals), or (4) Western diet with 10% of weight (g/kg) substituted with apple pomace (Western/AP). AIN diets were adjusted to be isocaloric (3.7–3.8 kcal/g) and Western diets were adjusted to be isocaloric (4.7 kcal/g), resulting in different types rather than amounts of simple and complex carbohydrates. Detailed ingredient composition of experimental diets is provided in Table 1. Locally sourced apple pomace was provided by Swilled Dog Hard Cider Company (Franklin, WV, USA) and nutrient composition analysis was performed by Medallion Laboratories (Minneapolis, MN, USA) (Table 2). Total polyphenols in apple pomace and treatment diets were determined while using the Folin-Ciocalteu method [21]. Diets were stored at −20 °C until fed. Rats were provided *ad libitum* access to their assigned diets and deionized distilled water (ddH_2_O) throughout the eight weeks study duration. At baseline (day 1) and final (end of eight weeks), rats were individually housed in a metabolic cage for 24 h to collect feces. Feces was weighed and dried. Food intake was measured and assigned diets were replaced every other day while ddH_2_O was replaced weekly. Rats were fasted overnight then euthanized by carbon dioxide inhalation. The liver was excised, perfused with 0.7% saline solution, weighed, and then flash frozen in liquid nitrogen. Rat livers were stored at −80 °C until analyzed.

### 2.2. Liver Total Lipid and Triglyceride Content

Lipid extraction was performed according to Bligh and Dyer [22]. Briefly, 1g of liver tissue was homogenized in Tris/EDTA buffer (pH 7.4). To quantify fatty acids, 50 µL of nonadecanoic acid (19:0) was added as a standard during the initial weighing of the samples. A chloroform: methanol:acetic acid (2:1:0.15, *v*/*v*/*v*) solution was added to liver samples, centrifuged at 900× *g* for 10 min at 10 °C, and the bottom chloroform layer collected. The collected chloroform layer was mixed with chloroform: methanol (4:1, *v*/*v*) and centrifuged at 900× *g* at 10 °C for 10 min. The chloroform layer was then collected and filtered. Extracted lipids were dried under nitrogen gas. Total lipid content in the liver was gravimetrically determined.

Liver triglyceride content was determined using a commercially available triglyceride colorimetric assay kit (Cayman Chemicals, Ann Arbor, MI, USA). Briefly, liver tissue (400 mg) was homogenized using assay standard diluent (2 mL). Tissue homogenate was centrifuged at 10,000× *g* for 10 min at 4 °C. Supernatant was collected and diluted 1:5 in assay standard diluent. Hepatic samples (10 µL) and lipase enzyme solution (150 µL) were added to a 96-well cell culture plate and then incubated for 15 min. Hepatic sample absorbance was measured at 540 nm using a BioTek Epoch microplate spectrophotometer (Winooski, VT, USA). All samples were performed in duplicate. The inter-assay coefficient of variation was 15.16%.

### 2.3. Diet and Liver Fatty Acid Composition

Following lipid extraction, diet and liver tissue samples were transmethylated according to the method described by Fritsche and Johnston [23]. Briefly, fatty acids were methylated by adding 4% sulfuric acid in anhydrous methanol to the extracted lipid samples followed by incubation in a 90 °C water bath for 60 min. Samples were cooled to room temperature and ddH_2_O was added. Chloroform was then added to the methylated samples and centrifuged at 900× *g* for 10 min at 10 °C. The collected chloroform layer was filtered through anhydrous sodium sulfate to remove any remaining water. Fatty acid methyl esters (FAMEs) were dried under nitrogen gas and re-suspended in iso-octane.

FAMEs were analyzed by gas liquid chromatography (CP-3800; Varian, Walnut Creek, CA, USA) using an initial temperature of 140 °C held for 5 min and then increased 1 °C per min to a final temperature of 220 °C. A wall-coated open tubular fused silica capillary column (Varian, Walnut Creek, CA, USA) was used to separate FAME with CP-Sil 88 at the stationary phase. Nitrogen was used as the carrier gas and the total separation time was 56 min. Quantitative 37 Component FAMEs Sigma Mix (Supelco, Bellefonte, PA, USA) was used to identify fatty acids. Fatty acids were determined by retention time and quantified using peak ultra-counts. All samples were performed in duplicate and reported as % of total fatty acids.

### 2.4. Liver Histology

The left lateral liver lobe (*n* = 7–8) was removed and immediately fixed in 10% buffered formalin solution for histological evaluation. Tissues were dehydrated through a series of increasing ethanol concentrations (70–100% in ddH_2_O), then placed in xylene and embedded in paraffin. Sections (8 µm) from each block were stained with hematoxylin and eosin. All slides were analyzed under a Nikon TE 2000-S light microscope (Nikon Instruments, Melville, NY, USA) by three trained individuals who were blinded to diet treatments. Liver fat accumulation was graded using the classification described by Brunt, et al., where grade 0 is no evidence of fat vacuoles, grade 1 is evidence of fat vacuoles in <33% of hepatocytes, grade 2 is evidence of fat vacuoles in 33–66% of hepatocytes, and grade 3 is evidence of fat vacuoles in >66% of hepatocytes [24]. Images were captured using a PC interface with Q-Capture imaging software (Quantitative imaging Corporation, BC, Canada).

### 2.5. RNA Isolation and Gene Expression

Total RNA was extracted from frozen tissue (50 mg) using the Zymo Research mRNA Isolation Kit (Irvine, CA, USA) according to the manufacturer’s instruction for total RNA isolation. Isolated RNA integrity was visualized on a 1.5% agarose gel and then quantified by spectrophotometry (NanoDrop 100; Thermo Scientific, Waltham, MA, USA). Following DNase I treatment with TURBO DNA-free kit (Applied Biosystems, Foster City, CA, USA), total mRNA was amplified using the Superscript III First-Strand Synthesis System with oligo dT primers (Invitrogen, Carlsbad, CA, USA).

Real-time quantitative polymerase chain reaction (RT-qPCR) consisted of 2.5 µL of SYBR Green Master Mix (Applied Biosystems, Foster City, CA, USA), 1 µL of cDNA (diluted 1:10), 1 µL of forward and reverse primer solutions (10 µM each), and 0.5 µL of deionized distilled water for a total reaction volume of 5 µL. The thermal profile consisted of 50 °C for 2 min, 95 °C for 10 min, and then 40 cycles of 95 °C for 15 s and 60 °C for 1 min. A melt curve analysis was applied at the end of cycling. Primers were designed for ChREBP, SREBP-1c, sterol regulatory binding protein 2 (SREBP2), FAS, SCD-1, peroxisome proliferator-activated receptor-α (PPARα), peroxisome proliferator-activated receptor-γ (PPARγ), hormone sensitive lipase (HSL), microsomal triglyceride transfer protein (MTTP), and DGAT2, as well as for housekeeping genes, β-actin and glyceraldehyde 2-phosphate dehydrogenase (GAPDH) using the Primer3 program (Howard Hughes Medical Institute) and respective mRNA sequences that were obtained from the NCBI database. Forward and reverse primers for gene transcriptions can be found in Appendix A.

### 2.6. Serum Biochemical Measurements

Rats were fasted overnight and euthanized by carbon dioxide inhalation. Blood was collected by aorta puncture. Collected blood was centrifuged at 1500× *g* for 10 min at 4 °C to obtain serum. Serum samples were stored at −80 °C until analyzed. Serum measures of liver function included: alanine aminotransferase (ALT) and aspartate aminotransferase (AST). Values were determined enzymatically using a commercially available Vet-16 rotor and were quantified by a Hemagen Analyst automated spectrophotometer (Hemagen Diagnostics Inc., Columbia, MD, USA). AST: ALT ratio was determined by dividing AST values by ALT values.

Serum cholesterol, low-density lipoprotein-cholesterol (LDL-C)/VLDL, and high-density lipoprotein-cholesterol (HDL-C) were determined by commercially available fluorometric assay (Cell Biolabs, San Diego, CA, USA). Briefly, 200 µL precipitation reagent was added to 200 µL of serum and then centrifuged at 2000× *g* for 20 min. The supernatant containing HDL-C was removed and diluted to a final volume of 1:50. Pelleted portion containing LDL-C/VLDL was resuspended in reaction buffer and diluted to a final volume of 1:50. Serum samples were aliquoted onto a 96-well plate, incubated for 45 min, and measured at excitation of 570 nm and emission at 590 nm using a BioTek Epoch microplate spectrophotometer.

Serum triglycerides were determined by commercially available colorimetric assay (Cayman Chemical, Ann Arbor, MI, USA). Briefly, 10 µL of serum was aliquoted onto a 96-well plate and reaction was initiated with 150 µL of diluted enzyme mixture solution. The plate was incubated at room temperature for 15 min and measured at a wavelength of 540 nm using a BioTek Epoch microplate spectrophotometer. All of the samples were performed in duplicate. The intra-assay coefficient of variation was 15.6%.

### 2.7. Serum Total Bile Acid Concentration

Serum total bile acid content was determined using a commercially available bile acid colorimetric assay kit (Crystal Chem Inc., Elk Grove, IL, USA). Briefly, 20 µL of serum and 150 µL of standard reagent were added to a 96-well plate and incubated for 5 min at 37 °C. Absorbance was measured at 540 nm on a BioTek Epoch microplate spectrophotometer. A second standard reagent (30 µL) was then added to all wells, followed by a 5 min incubation at 37 °C and absorbance read again at 540 nm. Differences between absorbance were measured to determine the total serum bile acid concentration. All samples were performed in duplicate. The intra-assay coefficient of variation was 38.5%.

### 2.8. Statistics

Results are expressed as mean ± standard error of the mean (SEM). Gene expression was determined as a function of mRNA abundance (A), where A = 1/(gene of interest primer efficiency × Δ_CT (g.o.i.)_ —average housekeeping primer efficiency × Δ_CT (h.k.),_ where the product of efficiency and average of expression of β-actin was averaged with the product of efficiency and average of expression of GAPDH to determine the overall expression of the two housekeeping genes [25]. For gene expression data, each treatment group was log-transformed prior to statistical analysis.

Data was analyzed for normal distribution and homogeneity of variance prior to conducting a one-way analysis of variance (ANOVA) to determine the differences among dietary groups. *Post hoc* multiple comparison tests were performed on parametric data using Tukey’s test with differences considered to be significant at *p* = 0.05 and a tendency at *p* = 0.08. Histological scoring was analyzed using the chi-square test. All statistical analyses were performed using JMP 12.2 statistical software package (SAS Institute, Cary, NC, USA).

## 3. Results

### 3.1. Fatty Acid Composition of Diets

As shown in Table 1, dietary fat content was higher in Western diets than standard AIN diets. Fatty acid analysis (Table 3) showed that Western/AP diet had the highest palmitic acid (16:0). Both Western diets contained significantly higher palmitic acid, stearic acid (18:0), palmitoleic acid (16:1n 7), and oleic acid (18:1n 9) as compared to AIN diets. Essential fatty acids, linoleic acid (18:2n-6), and α-linoleic acid (18:3n-3) content were approximately seven-fold lower (*p* < 0.0001) in Western diets when compared to AIN diets. Arachidonic acid was higher (*p* < 0.0001) in Western diets as compared to AIN diets, which contained negligible amounts.

### 3.2. Food Intake, Body Weight and Tissue Weights

**Figure 1:**
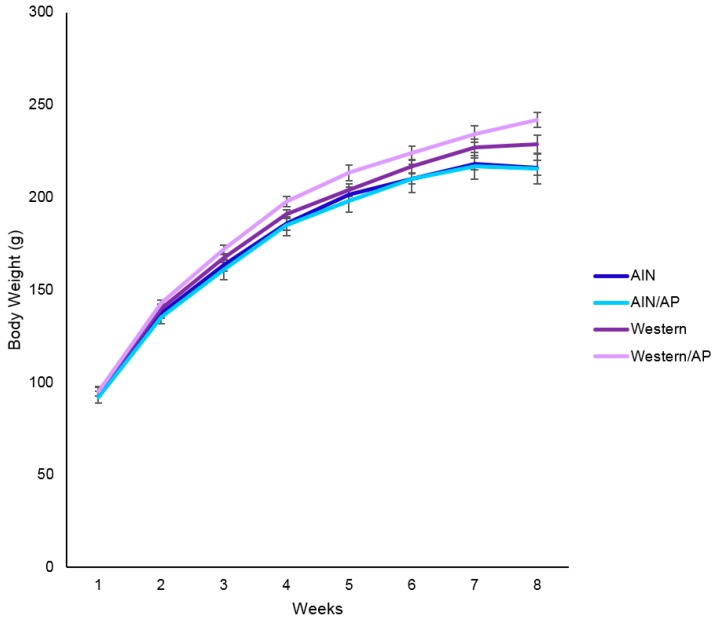
Growth curve of rats fed different diets containing apple pomace.

Figure 1 shows there was no significant differences in body weight among diet groups over eight weeks. As shown in Table 4, there was a tendency (*p* = 0.08) for higher final body weights for rats fed Western diets compared to AIN diets. Growing female rat fed Western diets consumed significantly more carbohydrates, fat, and total calories than rats fed standard rodent AIN diets. There were no statistically significant differences in amount of carbohydrates, fat, and total calories consumed by rats fed Western diet compared to Western/AP diet. There was a tendency (*p* = 0.08) for higher initial wet and dry fecal weights in rats fed Western as compared to AIN diets, but no significant differences in final fecal output among diet groups. There were no statistical differences observed in feed efficiency ratio among diet groups. Rats consuming Western diets had increased (*p* < 0.001) gonadal fat pad weights when compared to rats that were fed AIN diets. There were no statistically significant differences in gonadal fat pad weight in rats fed Western diet as compared to Western/AP diet. There were no statistically significant differences in absolute or relative liver weights among diet groups.

### 3.3. Liver Total Lipid and Triglyceride Content

**Figure 2:**
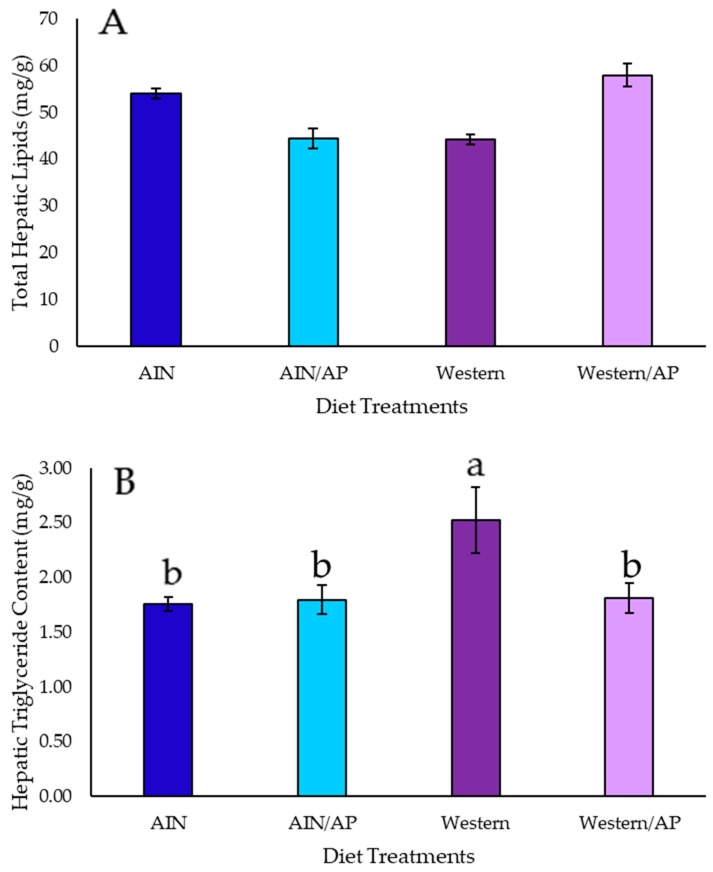
(**A**) Total hepatic lipid content (mg/g) and (**B**) Total hepatic triglyceride content (mg/g) of growing female rats consuming different diets substituted with apple pomace (10% *w*/*w*) following eight weeks of feeding. Different letters a and b indicate significant difference at *p* < 0.05 by one-way ANOVA followed by Tukey’s test.

Total lipids and triglyceride content in the liver were within the value range reported in previous studies of NAFLD [17]. There were no statistically significant differences in hepatic total lipid content among diet groups (Figure 2A). Rats that were fed Western diet had the highest (*p* = 0.01) hepatic triglyceride content. Rats fed Western/AP diet showed no significant differences in liver triglyceride content as compared to rats fed AIN diets (Figure 2B).

### 3.4. Liver Histology

**Figure 3:**
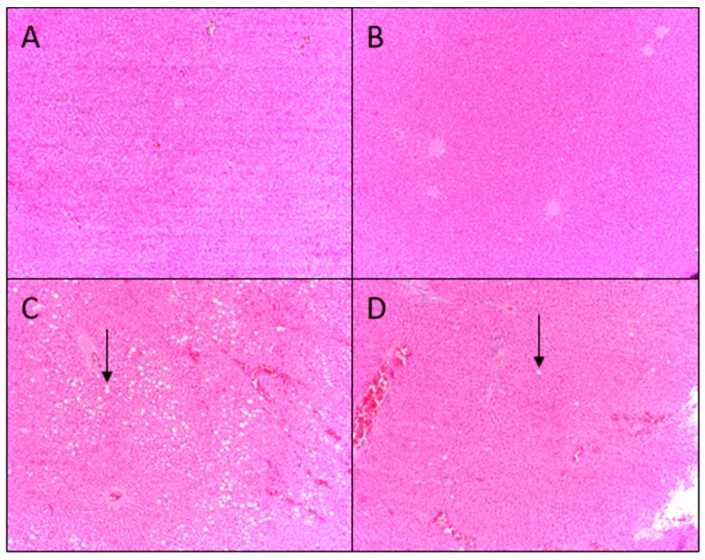
Representative histological staining images of livers of growing female rats consuming (**A**) AIN, (**B**) AIN/AP, (**C**) Western, or (**D**) Western/AP following eight weeks of feeding. Arrow indicates fat deposition. Scores analyzed by chi-square test at *p* < 0.05.

Based on liver histology, 14% of rats fed AIN diet had fat vacuoles in <33% of hepatocytes (Figure 3A) and 43% of rats fed AIN/AP diet had fat vacuoles in <33% of hepatocytes (Figure 3B). Higher hepatic fat infiltration was indicated by 25% of rats fed Western diet having fat vacuoles in 33–66% hepatocytes (Figure 3C), while 13% of rats fed Western/AP diet had fat vacuoles in 33–66% of hepatocytes (Figure 3D). There was an overall significant (*p* < 0.0001) difference among histology scores.

### 3.5. Liver Fatty Acid Composition

As shown in Table 5, rats that were fed Western diet had significantly higher hepatic palmitic acid content than rats fed AIN diets. While the Western/AP diet contained the highest amount of palmitic acid, liver content in rats fed Western/AP diet was only significantly higher when compared to rats fed AIN/AP diet. Western diets contained higher amounts of stearic acid and showed a tendency (*p* = 0.08) for higher hepatic stearic acid content than rats fed AIN diets. Both Western diets also contained higher amounts of palmitoleic and oleic acid than AIN diets. However, rats consuming Western diet had the highest hepatic palmitoleic acid content (*p* = 0.05). Rats fed Western diet, but not Western/AP diet had higher hepatic oleic acid content (*p* = 0.0005) compared to rats fed AIN diets. Rats consuming Western diets had significantly lower (*p* < 0.0001) hepatic linoleic and α-linolenic acid content when compared to rats consuming AIN diets, however no difference in hepatic arachidonic acid content was observed among all groups, despite negligible amounts in the AIN diets.

### 3.6. Hepatic Lipogenic Gene Expression

As shown in Table 6, hepatic DGAT2 gene expression was up-regulated (*p* < 0.01) in rats consuming Western diet compared to all diet groups. The Western/AP diet reverted hepatic DGAT2 gene expression to that found in rats fed AIN diets. There were no statistically significant differences in hepatic gene expression of ChREBP, SREBP-1c, SREBP-2, SCD-1, FAS, PPARα, PPARγ, HSL or MTTP among diet groups.

### 3.7. Serum Liver Enzymes, Cholesterol, Triglyceride, and Bile Acid Measurements

As shown in Table 7, there were no statistical significant differences in serum AST, ALT, AST:ALT ratio among diet groups. Serum triglycerides, VLDL/LDL-C, HDL-C, and total cholesterol were not significantly different among diet groups. Serum bile acid concentration was significantly higher in rats fed Western diet, but not Western/AP compared to rats fed AIN diets. 

## 4. Discussion

Rats that were provided Western diets ingested more (*p* < 0.0001) calories than rats provided standard AIN rodent diets and had greater (*p* < 0.0001) gonadal fat pad weight, although not heavier body weight. In addition to being associated with obesity, diets that are high in saturated fats and simple carbohydrates typified by Western diets have been associated with the development of NAFLD [26,27]. Liver steatosis has also been diagnosed in non-obese patients [8]. Replacing sugars in the diet, as well as increasing fruit consumption, is recommended [28]. However, studies have reported high fructose consumption induces NAFLD by stimulating hepatic DNL [17,26]. Among fruits popularly consumed in the USA, apples are considered to be particularly concentrated in fructose [2,29]. In the present study, nutrient analysis of apple pomace showed the major sugar was fructose (>30%).

Histological evaluation of liver tissue showed low fat infiltration (fat vacuoles in <33% of hepatocytes), with no significant increase in total lipid or triglyceride content in rats consuming AIN/AP as compared to AIN diet. Hepatic gene expression of DNL transcription factors and enzymes were not upregulated and there were no increases in end products: palmitic, stearic, palmitoleic, or oleic acid content in the liver of rats fed AIN/AP when compared to the AIN diet. Furthermore, there were no significant differences in serum ALT, AST, and ALT/AST ratio to indicate liver damage and dysfunction. Conversely, rats fed Western diets showed greater hepatic lipid accumulation, as indicated by fat vacuoles in 33–66% of hepatocytes. Rats fed Western diet showed 25% when compared to 14% of animals fed Western/AP having fat vacuoles in 33–66% of hepatocytes. Rats fed Western/AP had decreased (*p* = 0.04) hepatic triglyceride content as compared to rats fed Western diet, suggesting that substituting calories in the Western diet with 10% apple pomace attenuates hepatic triglyceride deposition.

Hepatic DNL is stimulated by FAS catalyzing synthesis of SFAs (i.e., palmitic and stearic acid). Our results showed no dietary effects on hepatic FAS gene expression. Yet, rats that were fed Western diet had higher (*p* = 0.0007) hepatic palmitic acid content when compared to rats fed either AIN diets. This may be explained by higher palmitic content of Western diets. Replacement with apple pomace in the Western/AP diet resulted in similar hepatic palmitic acid content to rats fed the AIN diet, but was higher than rats fed AIN/AP diet. In DNL, palmitic acid and stearic acid can be desaturated by the enzyme SCD-1 to MUFAs and palmitoleic and oleic acid, respectively. Despite no significant differences in dietary MUFA content between the Western and Western/AP diets, rats consuming Western diet had the highest (*p* = 0.05) liver palmitoleic acid content. Serum palmitoleic acid has been shown to be to be elevated in patients with liver disease and high serum VLDL [30,31]. Additionally, rats consuming a Western diet, but not Western/AP diet, had higher (*p* = 0.0005) hepatic oleic acid content as compared to rats consuming AIN diets. Studies have suggested that high oleic acid stimulates hepatic fat deposition, since oleic acid is the preferred substrate for hepatic triglyceride synthesis [32]. In a human clinical study, higher serum oleic acid was positively correlated to NAFLD [33]. In the present study, despite differences in liver MUFA content, gene expression of SCD-1 was not significantly different among diet groups. Differences in hepatic fat infiltration and fatty acid composition in the absence of changes in DNL gene expression may be due instead to diet influencing genes regulating lipolysis.

HSL catalyzes the conversion of diacylglycerols to monoacylglycerols in lipolysis [31]. PPARα and PPARγ have been suggested as a potential therapeutic target for NAFLD, as the upregulation of these transcription factors results in increased use of lipids for metabolism [34,35]. Therefore, gene expression of HSL, as well as transcription factors PPARα and PPARγ, were determined to assess whether increased lipolysis was responsible for the observed hepatoprotective effects of apple pomace. In the current study, the expression of genes regulating lipolysis were not significantly different among diet groups. Besides imbalanced DNL and lipolysis, altered hepatic lipid storage and transport has been suggested to be key to NAFLD [31,36]. Increased gene expression of DGAT2 has been reported to promote hepatic steatosis [37,38]. Reducing DGAT2 has also been identified as a therapeutic target for NAFLD [39]. In the current study, hepatic DGAT2 gene expression was upregulated (*p* = 0.002) nearly three-fold in rats that were fed a Western diet. The mechanism for higher triglyceride content in the liver of rats fed Western diet might be explained by the combination of upregulation of DGAT2 gene expression and increased MUFAs and palmitoleic and oleic acid content in the liver, since MUFAs have been shown to be preferentially used for triglyceride synthesis [32,40]. On the other hand, polyunsaturated fatty acids (PUFAs) have been reported to have anti-steatosis effects [33]. In our study, rats fed both Western diets had significantly lower hepatic PUFA (e.g., linoleic acid and α-linolenic acid) content than rats that were fed AIN diets. There was no difference in hepatic PUFA content in rats fed Western diet as compared to the Western/AP diet. Based on our results, MUFAs appeared to be the bioactive fatty acids inducing NAFLD in the Western diet.

Once synthesized triglycerides enter storage or secretory pools, hepatic MTTP regulates the packaging of triglycerides into VLDLs for transport into the circulation [41,42,43]. In our study, hepatic gene expression of MTTP was not significantly different among the diet groups. This was indicated by no significant changes in serum triglycerides and LDL-C/VLDL among diet groups. Overexpression of DGAT2 in mouse liver has been shown to increase liver triglyceride content, but not VLDL secretion [44]. Based on the results, greater total triglyceride accumulation in the liver of rats that were fed Western diet was due to increased triglyceride synthesis without a concomitant increase in the transport of triglycerides out of the liver. Accumulation of hepatic triglycerides without increasing circulating VLDLs may be due to the physical limitations of liver to export triglyceride-rich VLDL particles that exceed the diameter of the sinusoidal endothelia pores [45].

On the other hand, DGAT2 gene expression in the liver was not significantly upregulated in rats fed Western/AP diet, suggesting a potential therapeutic role of apple pomace in ameliorating Western diet induced NAFLD. Studies have suggested that bile represses hepatic triglyceride secretion, therefore reducing triglyceride accumulation [46]. Individuals with NAFLD have been reported to have higher serum bile acid [47]. Dietary fiber has been reported to decrease serum bile acids [48]. Individuals with NAFLD have been shown to consume less dietary fiber than healthy individuals [49]. Apple pomace contains a substantial amount of non-digestible carbohydrates, including: dietary fibers, pectin, and oligosaccharides [2]. In our study, serum bile acid concentration was higher in rats fed Western diet (*p* < 0.05), but not Western/AP diet as compared to rats fed AIN diets. Western diet was adjusted to remain isocaloric after substitution with apple pomace. All diets were adjusted to have 5% total fiber. No significant differences in body weight, fecal weights, and feed efficiency ratio suggested no differences in digestible energy among diet. However, differences in fiber type may be a potential mechanism. Studies have reported that dietary oligofructose decreases intra-hepatic triglycerides [50]. Hepatocytes that were isolated from non-digestible carbohydrate oligofructose-fed rats showed a reduced capacity to esterify palmitic acid [51]. In our study, rats fed Western diet, but not Western/AP, had higher (*p* = 0.0007) palmitic acid content in the liver when compared to rats fed the AIN diet. Consumption of fruits and dietary fiber have been shown to improve liver steatosis [49,52]. Therefore, as a fruit-based product that is high in dietary fiber, apple pomace could potentially improve dietary fiber consumption in individuals with hepatic steatosis and the risk of NAFLD.

A study of fiber-rich colloids that were isolated from apple pomace reported increased fecal excretion of bile acids with dietary fiber by fiber acting as a bile sequestrant to improve serum lipoproteins [53]. Another potential mechanism is the effect of soluble fibers on microbiota. Mice fed a 30% fat diet supplemented with 4% pectin modulated microbiota and increased short-chain fatty acid (SCFA) production resulting in a reduction in NAFLD [54]. However, extraction and purification of isolated ingredients from food can be technologically challenging and costly. Also, nutrients in foods often act synergistically. Young male rats that were fed a standard diet supplemented with 5 or 15% apple pomace for four weeks increased cecal SCFAs [55,56]. However, there has been a dearth of studies investigating the effects of apple pomace on lipid metabolism. Bobek, et al., focusing specifically on cholesterol metabolism in the liver, reported the beneficial effects of a 5% (*w*/*w*) apple pomace supplementation [57]. Another study reported diet-induced obese male Sprague-Dawley rats fed a high-fat diet (15% *w*/*w*) that was substituted with 10% (*w*/*w*) apple pomace for five weeks as compared to rats fed a high-fat diet resulted in significantly reduced liver triglyceride content, serum total triglycerides, total cholesterol, and LDL-C due to higher fecal triglyceride and cholesterol excretion [5]. In the present study, healthy growing female Sprague-Dawley rats fed the Western/AP diet attenuated hepatic fat infiltration and also attenuated elevated MUFA and triglyceride content induced by the Western diet. Additionally, elevated circulating bile acids was attenuated by apple pomace consumption. In contrast to the study on diet-induced obese male rats fed apple pomace, our study using female rats showed no improvement in serum lipoproteins [5]. Studies have shown that various types of diets that are used for developing NAFLD in experimental animals produce different effects [58]. In our study, Western diet induced hepatic steatosis was due to the dysregulation of hepatic triglyceride synthesis without changes in circulating lipoproteins. Similar hepatic effects were observed in other studies where the DGAT2 gene was overexpressed [44].

In summary, substituting calories with 10% apple pomace, despite added dietary fructose, did not promote liver steatosis in rats that were fed a standard AIN diet. Caloric substitution with fiber-rich apple pomace attenuated hepatic steatosis due to elevated hepatic MUFA content, higher circulating bile acids, and upregulated hepatic DGAT2 gene expression induced by a Western (high fat/high sugar) diet. Using a rat model, apple pomace consumption attenuated liver steatosis and had no detrimental effects on liver health. The abundance of apple pomace, currently a food processing waste by-product, has the potential to be re-purposed into a sustainable food product with beneficial health properties. Further mechanistic studies, preclinical, and human clinical research investigating apple pomace for human consumption and health can offer an environmental and economical solution for fruit waste that is generated by the industrial processing of apples.

## Figures and Tables

Histological steatosis scores for diet groups.

**Table:** 

Measurement	AIN	AIN/AP	Western	Western/AP	*p*-Value
Steatosis Grade					<0.0001
0	6	4	-	-	
1	1	3	6	7	
2	-	-	2	1	
3	-	-	-	-	

**Table 1:** Composition of rodent diets substituted with apple pomace (10% g/kg) fed to growing female rats.

Diet Groups ^1^				
	AIN	AIN/AP	Western	Western/AP
Ingredients (g/kg) ^1^				
Apple pomace	0.0	100.0	0.0	100.0
Corn Starch	397.486	392.086	63.36	57.96
Maltodextrin	132.0	132.0	60.0	60.0
Sucrose	100.0	43.9	340.0	283.9
Total Dietary Fiber	50.0	50.0	50.0	50.0
Insoluble Fiber ^2^	50.0	39.0	50.0	39.0
Soluble Fiber ^3^	0.0	11.0	0.0	11.0
Anhydrous Milkfat	0.0	0.0	210.0	210.0
Soybean Oil	70.0	68.7	20.0	18.7
Casein	200.0	196.0	195.0	191.0
L-Cystine	3.0	3.0	3.0	3.0
Vitamin Mix	10.0	10.0	12.5	12.5
Mineral Mix	35.0	35.0	43.0	43.0
Choline Bitartrate	2.5	2.5	3.1	3.1
TBHQ, antioxidant	0.014	0.014	0.04	0.04
Polyphenols	0.0015	0.0029	0.0008	0.0032
Macronutrients (% kcal)				
Protein	18.8	18.9	14.8	14.8
Fat	17.2	17.3	44.6	44.8
Carbohydrate	63.9	63.7	40.6	40.4
Calories (kcal/g)	3.8	3.7	4.7	4.7

^1^ Abbreviations: AIN, the American Institute of Nutrition; AP, apple pomace; TBHQ, tert-butylhydroquinone. ^2^ Insoluble fiber is cellulose. ^3^ Soluble fiber is mainly pectin [2].

**Table 2:** Composition of freeze-dried apple pomace substituted (10% g/kg) into diets fed to growing female rats.

Macronutrients (%)	
Protein	3.56
Fat	1.3
Carbohydrates	68.1
*Sugars (%)*	
Fructose	32.5
Glucose	9.77
Sucrose	13.9
Maltose	<0.1
Lactose	<0.1
*Dietary Fiber (%)*	
Insoluble Dietary Fiber	22.2
Soluble Dietary Fiber	11.0
Polyphenols (g/kg)	0.029
Calories (kcal/100 g)	387

**Table 3:** Fatty acid analysis of rodent diets substituted with apple pomace (10% g/kg).

Measurements	Treatments ^1^				
	AIN	AIN/AP	Western	Western/AP	*p*-Value
**SFAs**					
Palmitic acid (16:0)	11.36 ± 0.09 ^c^	11.14 ± 0.15 ^c^	32.19 ± 0.03 ^b^	32.92 ± 0.30 ^a^	<0.0001
Stearic acid (18:0)	3.56 ± 0.25 ^b^	3.72 ± 0.06 ^b^	9.94 ± 0.11 ^a^	10.24 ± 0.06 ^a^	<0.0001
**MUFAs**					
Palmitoleic acid (16:1n-7)	0 ± 0.00 ^b^	0 ± 0.00 ^b^	1.44 ± 0.01 ^a^	1.44 ± 0.02 ^a^	<0.0001
Oleic acid (18:1n-9)	19.09 ± 0.10 ^b^	18.35 ± 0.33 ^b^	22.96 ± 0.11 ^a^	22.95 ± 0.17 ^a^	<0.0001
**PUFAs**					
Linoleic acid (18:2 n-6)	50.12 ± 0.55 ^a^	51.41 ± 2.41 ^a^	6.99 ± 0.09 ^b^	7.04 ± 0.06 ^b^	<0.0001
α-linolenic acid (18:3 n-3)	7.08 ± 0.13 ^a^	7.13 ± 0.70 ^a^	1.04 ± 0.01 ^b^	1.05 ± 0.02 ^b^	<0.0001
Arachidonic acid (20:4 n-6)	0 ± 0.00 ^b^	0 ± 0.00 ^b^	0.13 ± 0.00 ^a^	0.14 ± 0.00 ^a^	<0.0001

Values expressed as mean ± standard error of the mean (SEM, *n* = 5 samples/group). Different superscript letters a, b, and c within. The same row indicates significant difference at *p* < 0.05 by one-way ANOVA followed by Tukey’s test. Abbreviations: MUFAs, monounsaturated fatty acids; PUFAs, polyunsaturated fatty, acids; SFAs, saturated fatty acids.

**Table 4:** Effect of consumption of different diets substituted with apple pomace (10% g/kg) by growing female rats on caloric intake, body weight, and liver weight following eight weeks of feeding.

Measurements	Treatments ^1^				
	AIN	AIN/AP	Western	Western/AP	*p*-Value
Caloric intake (kcal)	2946 ± 85 ^b^	2757 ± 62 ^b^	3373 ± 71 ^a^	3443 ± 134 ^a^	<0.0001
CHO intake (kcal)	1769 ± 54 ^a^	1708 ± 39 ^a^	1354 ± 29 ^b^	1347 ± 54 ^b^	<0.0001
Fat intake (kcal)	476 ± 15 ^b^	464 ± 11 ^b^	1487 ± 32 ^a^	1494 ± 60 ^a^	<0.0001
Initial bwt (g)	95 ± 3	92 ± 3	95 ± 3	95 ± 3	0.80
Final bwt (g)	216 ± 4	216 ± 8	228 ± 5	234 ± 5	0.08
Total bwt gain (g)	121 ± 4	124 ± 7	133 ± 6	138 ± 6	0.17
Wet Initial Fecal Weight (g)	0.82 ± 0.06 ^b^	0.75 ± 0.11 ^b^	1.30 ± 0.23 ^a^	1.14 ± 0.13 ^a,b^	0.06
Dry Initial Fecal Weight (g)	0.67 ± 0.06	0.54 ± 0.14	1.269 ± 0.27	0.75 ± 0.21	0.08
Wet Final Fecal Weight (g)	0.30 ± 0.18 ^b^	0.28 ± 0.16 ^b^	0.91 ± 0.34 ^a^	0.63 ± 0.23 ^a,b^	0.03
Dry Final Fecal Weight (g)	0.23 ± 0.13	0.19 ± 0.10	0.78 ± 0.27	0.50 ± 0.16	0.13
Feed Efficiency Ratio	0.17 ± 0.01	0.17 ± 0.01	0.18 ± 0.01	0.18 ± 0.01	0.13
Gonadal fat pad weight (g)	4.12 ± 0.26 ^b^	3.46 ± 0.44 ^b^	5.87 ± 0.24 ^a^	5.96 ± 0.23 ^a^	<0.0001
Liver weight (g)	7.50 ± 0.24	7.44 ± 0.37	8.05 ± 0.30	7.98 ± 0.24	0.35
Relative liver weight (mg/g bwt)	3.47 ± 0.078	3.45 ± 0.068	3.52 ± 0.067	3.41 ± 0.048	0.69

^1^ Values expressed as mean ± SEM (*n* = 6–8 rats/group). Different superscript letters a and b within the same row. Indicate significant difference at *p* < 0.05 by one-way ANOVA followed by Tukey’s test. Abbreviations: Bwt, body weight; CHO, carbohydrate.

**Table 5:** Effect of consumption of different diets substituted with apple pomace (10% g/kg) by growing female rats on hepatic fatty acid composition following 8 weeks of feeding.

Measurements (%)	Treatments ^1^				
	AIN	AIN/AP	Western	Western/AP	*p*-Value
**SFAs**					
Palmitic acid (16:0)	19.10 ± 0.57 ^b,c^	18.40 ± 0.53 ^c^	21.44 ± 0.53 ^a^	21.08 ± 0.53 ^a,b^	0.0007
Stearic acid (18:0)	14.46 ± 0.65	14.06 ± 0.60	14.83 ± 0.83	16.28 ± 0.60	0.08
**MUFAs**					
Palmitoleic acid (16:1n-7)	0.57 ± 0.25 ^b^	0.81 ± 0.25 ^b^	1.61 ± 0.23 ^a^	0.76 ± 0.25 ^b^	0.05
Oleic acid (18:1n-9)	11.25 ± 1.65 ^b^	10.72 ± 1.39 ^b^	19.55 ± 1.39 ^a^	15.95 ± 1.30 ^a,b^	0.0005
**PUFAs**					
Linoleic acid (18:2n-6)	22.44 ± 1.09 ^a^	25.12 ± 1.09 ^a^	9.28 ± 1.09 ^b^	8.38 ± 1.09 ^b^	<0.0001
α-linoleic acid (18:3n-3)	1.04 ± 0.14 ^a^	1.28 ± 0.15 ^a^	0.23 ± 0.14 ^b^	0.22 ± 0.14 ^b^	<0.0001
Arachidonic acid (20:4n-6)	13.26 ± 1.00	11.99 ± 1.61	13.20 ± 1.00	14.56 ± 1.00	0.37

^1^ Values expressed as mean ± SEM (*n* = 6–8 rats/group). Different superscript letters a, b, and c within the same row indicate significant difference at *p* < 0.05 by one-way ANOVA followed by Tukey’s test. Abbreviations: MUFAs, monounsaturated fatty acids; PUFAs, polyunsaturated fatty acids; SFAs, saturated fatty acids.

**Table 6:** Effect of consumption of different diets substituted with apple pomace (10% g/kg) by growing female rats on hepatic lipid metabolism gene expression following 8 weeks of feeding.

Measurements	Treatments ^1^				
	AIN	AIN/AP	Western	Western/AP	*p*-Value
**Lipogenesis**					
ChREBP	0.074 ± 0.004	0.069 ± 0.006	0.078 ± 0.006	0.077 ± 0.009	0.76
SREBP-1c	0.118 ± 0.027	0.132 ± 0.041	0.136 ± 0.038	0.117 ± 0.031	0.11
SREBP-2	0.088 ± 0.020	0.080 ± 0.012	0.083 ± 0.017	0.092 ± 0.026	0.92
FAS	0.116 ± 0.034	0.096 ± 0.015	0.121 ± 0.028	0.156 ± 0.075	0.15
SCD-1	0.234 ± 0.084	0.216 ± 0.036	0.223 ± 0.073	0.309 ± 0.080	0.13
**Lipolysis**					
PPARα	0.088 ± 0.016	0.099 ± 0.013	0.098 ± 0.027	0.076 ± 0.012	0.74
PPARγ	0.054 ± 0.021	0.053 ± 0.013	0.053 ± 0.016	0.052 ± 0.026	0.99
HSL	0.085 ± 0.019	0.087 ± 0.011	0.084 ± 0.023	0.074 ± 0.027	0.89
**Storage and Transport**					
DGAT2	0.171 ± 0.020 ^b^	0.151 ± 0.025 ^b^	0.617 ± 0.161 ^a^	0.213 ± 0.039 ^b^	0.002
MTTP	0.145 ± 0.026	0.090 ± 0.008	0.164 ± 0.044	0.151 ± 0.035	0.43

^1^ Values expressed as mean ± SEM (*n* = 6–8 rats/group) of transcript abundance (A) of gene of interest relative to housekeeping genes β-actin and GAPDH. Different superscript letters a and b within the same row indicate significant difference at *p* < 0.05 by one-way ANOVA followed by Tukey’s test. Abbreviations: ChREBP, carbohydrate element response binding protein; DGAT2, diacylglycerol O-acyltransferase 2; FAS, fatty acid synthase; HSL, hormone sensitive lipase; MTTP, microsomal triglyceride transfer protein; PPARα, peroxisome proliferator-activated receptor alpha; PPARγ, peroxisome proliferator-activated receptor gamma; SCD-1, stearoyl-CoA desaturase-1; SREBP-1c, sterol regulatory binding protein-1c; SREBP-2, sterol regulatory binding protein-2.

**Table 7:** Effect of consumption of different diets substituted with apple pomace (10% g/kg) by growing female rats on serum measurements of liver function enzymes, cholesterol, and bile acids following 8 weeks of feeding.

Serum Measurements	AIN	AIN/AP	Western	Western/AP	*p*-Value
AST (U/L)	129.48 ± 52.86	212.50 ± 37.86	283.63 ± 45.30	259.67 ± 48.96	0.69
ALT (U/L)	107.63 ± 19.59	118.71 ± 43.60	94.5 ± 12.58	133.5 ± 30.59	0.78
AST:ALT ratio	3.16 ± 0.45	2.79 ± 0.28	2.97 ± 0.26	2.84 ± 0.17	0.83
VLDL/LDL-C (mg/dL)	41.59 ± 4.17	41.74 ± 2.38	40.42 ± 6.44	41.29 ± 6.74	0.99
HDL-C (mg/dL)	17.29 ± 2.18	18.95 ± 1.97	18.21 ± 2.13	21.85 ± 1.82	0.43
Total Cholesterol (mg/dL)	58.89 ± 3.43	60.38 ± 3.38	59.80 ± 5.69	57.98 ± 7.85	0.99
Triglyceride (mg/dL)	55.81 ± 8.17	47.39 ± 9.04	58.64 ± 9.38	50.04 ± 8.81	0.84
Total Bile Acids (µmol/L)	30.00 ± 3.13 ^b^	28.80 ± 7.69 ^b^	54.13 ± 7.96 ^a^	31.52 ± 3.69 ^a,b^	0.02

^1^ Values expressed as mean ± SEM of *n* = 6–8 rats/group. Different superscript letters a and b within the same row indicate significant difference at *p* < 0.05 by one-way ANOVA followed by Tukey’s test. Abbreviations: AST, aspartate aminotransferase; ALT, alanine aminotransferase; VLDL, very-low density lipoprotein, LDL-C, low density lipoprotein-cholesterol; HDL-C, high density lipoprotein-cholesterol.

## Appendix A

**Table A1:** List pf primers used for RT-qPCR analysis.

Gene	NCBI Accession Number	Forward Primer	Reverse Primer
ChREBP	NM_133552.1	5′ CCTTAATGGGATGGTGTCT 3′	5′ AGAGCAGGGCAGGGAACAGTA 3′
SREBP-1c	NM_001276707.1	5′ GCCTGCTTGGCTCTTCTCT 3′	5′ GCTTGTTTGCGATGTCTCC 3′
SREBP-2	NM_001033694.1	5′ TGATGCCGACTCTACTGCTG 3′	5′ CACGAAGACGCTCAAGACAA 3′
FAS	NM_017332.1	5′ GCTGCTACAAACAGCACCATC 3′	5′ TCCACTGACTCTTCACAGACCA 3′
SCD-1	NM_139192.2	5′ TTCGCCACTGACTTGCTATG 3′	5′ CAGGAGGTTCTTGGGATGATT 3′
PPARα	NM_013196.1	5′ ATGAACAAAGACGGGATGCT 3′	5′ AGGAACTCTCGGGTGATGAA 3′
PPARγ	NM_013124.3	5′ GCAAAGACAACAGACAAATCACC 3′	5′ CGAAACTGGCACCCTTGA 3′
HSL	NM_012859.1	5′ TCTCCAAGTGTGTAGCGCCTA 3′	5′ AACTCCAGGAAGGAGTTGAGC 3′
MTTP	NM_001107727.1	5′ TCAGGTGCTGTGTATTCTGCT 3′	5′ CGTGTTCCACTTCCGTTCT 3′
DGAT2	NM_001012345.1	5′ CACCCTCCTGTGTATTCTGCT 3′	5′ TTTGGCTGTTCTTGTTTCCA 3′
Β-actin	NM_031144.2	5′ TTGCTGACAGGATGCACAAG 3′	5′ CAGTGAGGCCAGGATAGAGC 3′
GAPDH	NM_017008.4	5′ TCAAGAAGGTGGTGAAGCAG 3′	5′ CCTCAGTGTAGCCCAGGATG 3′

Abbreviations: Abbreviations: ChREBP, carbohydrate element response binding protein; DGAT2, diacylglycerol O-acyltransferase 2; FAS, fatty acid synthase; HSL, hormone sensitive lipase; MTTP, microsomal triglyceride transfer protein; PPARα, peroxisome proliferator-activated receptor alpha; PPARγ, peroxisome proliferator-activated receptor gamma; SCD-1, stearoyl-CoA desaturase-1; SREBP-1c, sterol regulatory binding protein-1c; SREBP-2, sterol regulatory binding protein-2.
